# Ductile B2 Intermetallics‐Driven Strength‐Ductility Synergy in Heterolaminated Multi‐Principal Element Alloys

**DOI:** 10.1002/advs.76697

**Published:** 2026-07-24

**Authors:** Lu Yang, Feilong Jiang, Qiming Zhuang, Dingshan Liang, Jiasi Luo, Kangjie Chu, Junhua Luan, Zengbao Jiao, Robert O. Ritchie, Fuzeng Ren

**Affiliations:** ^1^ Department of Materials Science and Engineering Southern University of Science and Technology Shenzhen Guangdong China; ^2^ Department of Materials Science and Engineering City University of Hong Kong Hong Kong China; ^3^ Department of Mechanical Engineering The Hong Kong Polytechnic University Hong Kong China; ^4^ Department of Materials Science and Engineering University of California Berkeley California USA

**Keywords:** ductile B2 intermetallics, heterogeneous structure, multi‐principal element alloy, strain hardening, strength and ductility

## Abstract

Conventional intermetallic‐strengthened alloys invariably suffer from a strength–ductility trade‐off, as brittle η, σ, and µ phases precipitate at interfaces and trigger premature failure. Here, we overturn this paradigm in a multi‐principal element alloy (MPEA) by deliberately engineering its B2 intermetallic phase to act not as a crack‐initiator, but as a ductile, load‐bearing constituent. Through intrinsic phase toughening enabled by multicomponent chemical complexity, pre‐existing dislocations, and local compositional fluctuations, we demonstrate that the B2 phase can sustain continuous dislocation glide and multiplication. Simultaneously, we impose a heterogeneous laminated architecture of face‐centered cubic (FCC) and B2 domains, which promotes strain delocalization and redistributes stress to suppress interface cracking. The resulting synergy culminates in an exceptional combination of mechanical properties at room temperature: a yield strength of 1.24 GPa, an ultimate tensile strength of 1.57 GPa, and 20% uniform elongation, along with a high strain hardening rate exceeding 3 GPa across a wide strain range. These findings establish a new design strategy through integrating ductile intermetallic phases with mesoscale heterogeneity to overcome the long‐standing strength‐ductility trade‐off in structural materials.

## Graphical abstract

Intrinsic phase toughening transforms a brittle B2 intermetallic into a plastically deformable, load‐bearing phase, while a heterogeneous laminated architecture suppresses strain localization and interface cracking. The synergy between ductile intermetallics and mesoscale heterogeneity establishes a general design strategy for breaking the long‐standing strength–ductility trade‐off in structural materials.

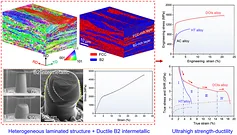

## 1 Introduction

Achieving the concurrent realization of high strength and large ductility remains a central challenge in structural alloy design [1, 2]. This well‐known strength–ductility trade‐off, rooted in the fundamentally antagonistic roles of mechanisms that promote strength versus those that accommodate plasticity, continues to limit the performance envelope of advanced metallic materials [3]. Multi‐principal element alloys (MPEAs) have emerged as a fertile platform for addressing this challenge. Owing to their high configurational entropy and severe lattice distortion, MPEAs display attractive combinations of properties, including high fracture toughness over broad temperature ranges [4], outstanding corrosion and oxidation resistance [5], and notable irradiation tolerance [6]. Nevertheless, single‐phase face‐centered cubic (FCC) MPEAs, while highly ductile, generally lack the yield strength necessary for demanding load‐bearing applications [7]. Although deformation‐induced twinning [4] and stress‐assisted martensitic transformations [8] can provide supplementary strengthening, these mechanisms alone often prove inadequate for achieving the ultrahigh strength levels while maintaining substantial ductility.

Precipitation strengthening offers a pathway to ultrahigh strength, but conventional intermetallic precipitates—such as η, σ, and µ phases—are typically non‐shearable, generating dislocation pile‐ups and stress concentrations at interfaces that promote early fracture [9, 10]. Coherent L1_2_ precipitates within FCC MPEAs have demonstrated a unique ability to raise strength without severely impairing ductility, owing to their shearable nature and low interfacial energy [11, 12]. In contrast, B2‐ordered intermetallics, typified by NiAl, are intrinsically brittle due to their limited slip systems and strong directional bonding [13]. Although nanometer‐scale B2 precipitates at low volume fractions can provide appreciable strengthening [1, 14, 15], increasing their fraction invariably leads to embrittlement [14]. This reflects the long‐standing assumption that B2 precipitates embedded in an FCC matrix are non‐shearable and primarily deform by Orowan looping, which inherently produces high interfacial stresses and reduces ductility [16].

Several strategies have been explored to reduce this trade‐off, including refining precipitate dispersion [17], activating additional slip systems [18], and, more recently, enabling the shearing of B2 nanoprecipitates. A landmark study by Wang et al. [13] demonstrated direct dislocation cutting of typically brittle B2 intermetallics in a lightweight compositionally complex steel during cryogenic tension. This mechanism, facilitated by an exceptionally high matrix friction stress (> 1 GPa) arising from local chemical ordering and severe solid‐solution strengthening, enables sequential precipitate shearing, thereby providing both strong strengthening and substantial strain hardening while maintaining ductility. This represents a paradigm shift from conventional Orowan looping to B2 particle shearing. Beyond monotonic deformation, B2 phases also contribute to notable back‐stress hardening in FCC/B2 MPEAs [19], and their phase‐transformable characteristics can significantly enhance fatigue resistance by reducing stress concentrations and delaying crack initiation [20]. Nonetheless, these mechanisms are typically effective only at low to moderate B2 fractions or under extreme conditions (e.g., cryogenic temperatures) and remain difficult to sustain in bulk structures with high B2 content. Therefore, a key unresolved challenge is how to exploit the high intrinsic strength of B2 intermetallics at elevated phase fractions without sacrificing damage tolerance. Addressing this challenge motivates the present work, which introduces a new design strategy that integrates intrinsic phase toughening with mesoscale architectural control to overcome the limitations of conventional B2‐based precipitation strengthening.

In parallel, heterostructure engineering has emerged as a complementary paradigm for bypassing the monolithic strength–ductility limit. By combining domains with distinct strength and strain‐hardening behaviors, heterogeneous architectures exploit hetero‐deformation‐induced (HDI) hardening from strain gradients and the associated storage of geometrically necessary dislocations (GNDs). For MPEAs, this concept has been realized in gradient structures [21], bimodal grain structures [22], hierarchical arrangements [23], and heterogeneous lamellae [24]. For example, Al_0.1_CoCrFeNi with a coarse/fine grain mixture achieved a yield strength of 711 MPa with 30.3% uniform elongation, while cyclic torsion‐induced gradient dislocation structures tripled yield strength to 539 MPa without loss of ductility [25]. These heterogeneous microstructures, featuring variations in grain size or dislocation density across different length scales, primarily rely on strain gradients that accumulate GNDs at domain boundaries. However, the strength ceiling for such heterostructures typically plateaus near ∼1 GPa, reflecting the limitations of GND storage in coarse domains and strain partitioning in finer domains. This plateau underscores the potential value of integrating secondary strengthening phases—such as B2 intermetallics—into a heterogeneous design.

Here, we demonstrate a dual‐strategy design in a Ni_2_FeVAl_0.5_ MPEA that transforms B2 intermetallics from brittle liabilities into ductile, load‐bearing constituents. First, intrinsic phase toughening—via multicomponent chemical complexity, tailored pre‐existing dislocations, and local compositional heterogeneity—activates multiple slip systems in B2 domains, enabling sustained dislocation glide. Second, a heterogeneous laminated architecture spatially partitions FCC and B2 regions, enabling strain delocalization and stress redistribution that suppress interfacial decohesion. The synergy of these mechanisms yields a remarkable property combination at room temperature: 1.24 GPa yield strength, 1.57 GPa ultimate tensile strength, and 20% uniform elongation. This work reframes the role of intermetallics in alloy design, establishing a pathway to damage‐tolerant, ultrahigh‐strength materials by coupling deformable intermetallic phases with mesoscale architectural heterogeneity.

## 2 Results

### 2.1 Microstructures

The alloy containing ductile, ordered intermetallics (B2 phase)—hereafter denoted the DOIs alloy—was produced by severe cold rolling followed by annealing at 850°C (see Supplementary Text and Experimental Section). X‐ray diffraction (XRD) (Figure 1a) confirms a duplex microstructure of FCC and B2 phases, in excellent agreement with Thermo‐Calc predictions (Figure S1a). Extrapolation of the peak positions yields lattice parameters of 3.598 Å for the FCC phase and 2.882 Å for the B2 phase. Electron backscatter diffraction (EBSD) inverse pole figure and phase maps (Figure 1b,c) reveal a hierarchical laminated architecture: alternating equiaxed, fully recrystallized FCC‐rich layers (∼0.94 µm average grain size) and lath‐shaped B2‐rich layers showing partial recrystallization. Kernel average misorientation (KAM) mapping (Figure S2) indicates elevated local misorientations within the B2 domains, reflecting abundant residual strain. Within the FCC‐laminated regions, nanoscale B2 precipitates (∼659 nm average) are uniformly dispersed, whereas irregular FCC grains are found in the B2 layers (Figure S3). Quantitative EBSD analysis reveals that the DOIs alloy contains a high‐volume fraction of B2 intermetallics—52 vol.%. High‐angle annular dark‐field scanning transmission electron microscopy (HAADF‐STEM) (Figure 1d) shows that the B2 intermetallics are enriched in Ni and Al. Quantitative energy‐dispersive X‐ray spectroscopy (EDS) analysis indicates that the B2 intermetallics contain 44.3 at.% Ni, 24.5 at.% Al, 17.2 at.% Fe and 14.0 at.% V. In contrast, the FCC phase is enriched in Ni, Fe, and V, while containing a relatively lower concentration of Al. Quantitative EDS analysis of the FCC regions indicates an average composition of approximately 42.9 at.% Ni, 28.7 at.% Fe, 22.8 at.% V, and 5.6 at.% Al. The selected area electron diffraction (SAED) pattern along [001] confirms the ordered BCC structure of the B2 intermetallics. Accordingly, we identify the hard phase as a multicomponent Ni(Al, Fe, V) B2 intermetallic, distinct from the conventional binary NiAl.

**FIGURE 1:**
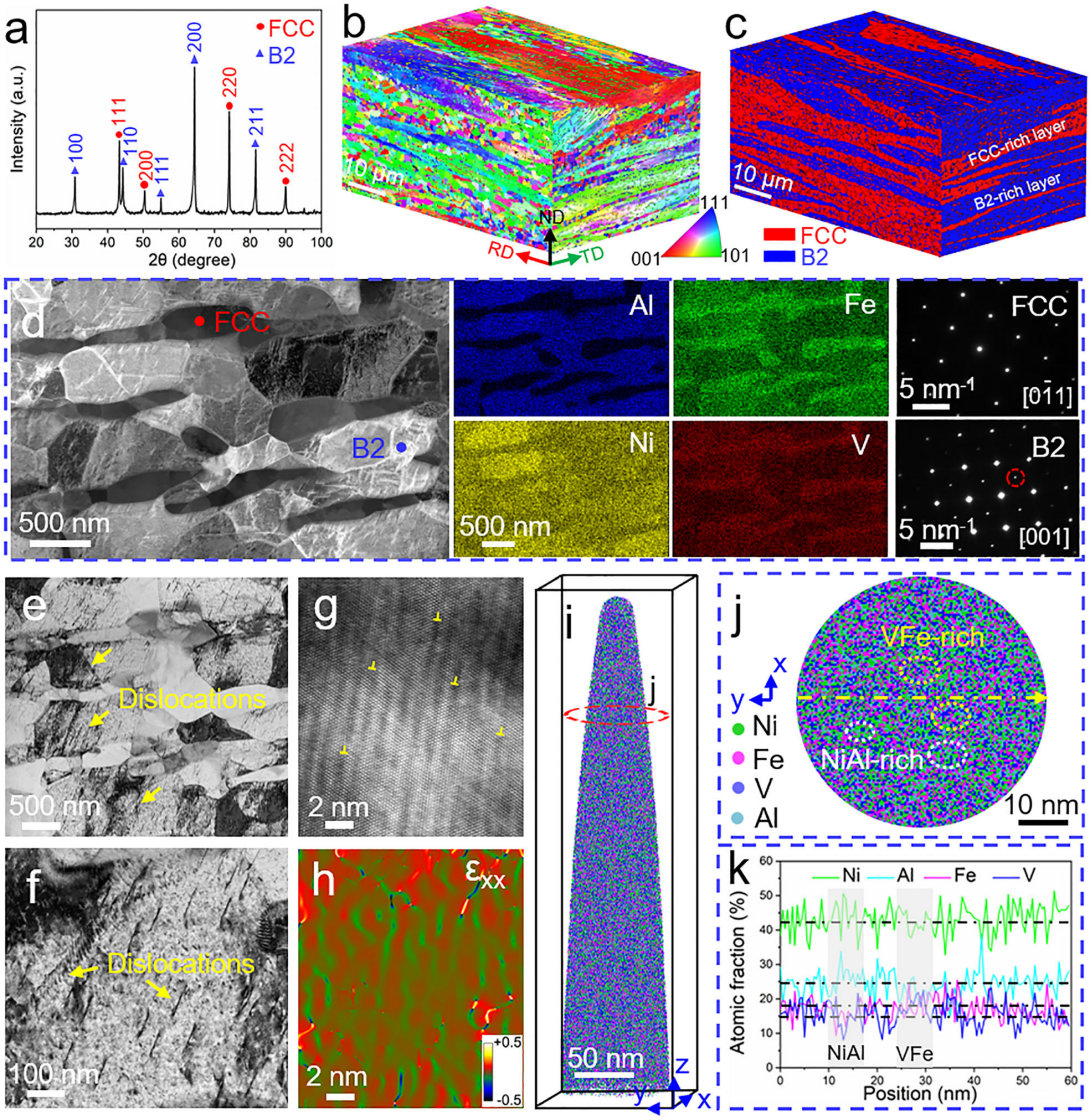
Microstructure of the ductile B2 intermetallics‐strengthened Ni_2_FeVAl_0.5_ alloy. (a) XRD pattern showing duplex FCC and B2 peaks. (b) EBSD inverse pole figure map. (c) EBSD phase map distinguishing FCC (red) and B2 (blue) domains. (d) HAADF‐STEM and corresponding EDS elemental maps of Al, Fe, Ni, and V. (e,f) Bright‐field TEM images revealing a high density of dislocations in B2 intermetallics. (g,h) High‐resolution TEM image and corresponding GPA strain map (in‐plane symmetric strain tensor, ε_xx_) of the B2 intermetallics along the $\left[\bar{1}11\right]$ zone axis. (i) APT three‐dimensional reconstruction map. (j) High‐resolution atom map on the x‐y plane. (k) Line scan composition profiles along the yellow dashed line in (j), indicating local chemical fluctuation in B2.

Bright‐field TEM images (Figure 1e,f) show a high density of residual dislocations (1.08×10^14^ m^−2^) in the B2 intermetallics, measured by the line intercept method [26], in stark contrast to the nearly dislocation‐free FCC regions (with detailed microstructures of the FCC regions shown in Figure S4). High‐resolution TEM along the [$\bar{1}11$] zone axis (Figure 1g) exposes clear dislocation lines within the B2 domains. Geometric phase analysis uncovers pronounced atomic‐scale lattice strains localized around these dislocations, indicative of strong barriers to dislocation motion and sites of enhanced work hardening, as shown in Figure 1h. The spatial variation of each element in the B2 intermetallics was further revealed by atom probe tomography (APT). Element distribution in Figure 1i,j demonstrates significant compositional heterogeneity within the B2 domains. Driven by the strong Ni–Al affinity (Δ*H*_mix_ = −22 kJ/mol [27]), NiAl‐rich and VFe‐rich regions form over length‐scales up to 10 nm. Line scan profiles (Figure 1k) reveal local deviations of ±9 at.% from the nominal composition, resembling the compositional undulations reported in single‐phase nanocrystalline NiCo alloys [28]. The presence of these chemical fluctuations is further corroborated by frequency distribution analysis (Figure S5), which shows a significant deviation of the elemental compositions from the ideal binomial distribution for a random solid solution.

### 2.2 Mechanical Response

To validate our design, we probed the microscale mechanical response of the individual single‐FCC phase and B2 intermetallics in the DOIs alloy using micropillar compression (Movies S1 and S2). Figure 2 compares the stress–strain curves and post‐deformation pillar morphologies for each phase. The FCC pillars yield at 1.18 ± 0.12 GPa and exhibit intermittent load drops in the stress–strain curve (Figure 2a). In striking contrast, the B2 pillars display continuous strain hardening; they yield at an average stress of 1.95 ± 0.18 GPa and strengthen steadily to 4.56 GPa at 30% strain, with no evidence of load drops (Figure 2b). Moreover, snaps of the FCC phase and B2 intermetallics at progressive strain were displayed in Figure 2c. The FCC pillars exhibit successive slip‐band formation during compression (Figure 2c,d). Unexpectedly, the B2 pillars deform homogeneously with only minor buckling near the top surface, indicating high plasticity (Figure 2c–e). These results confirm that our engineered B2 intermetallics are intrinsically ductile and capable of substantial plastic deformation—unlike traditional brittle intermetallics.

**FIGURE 2:**
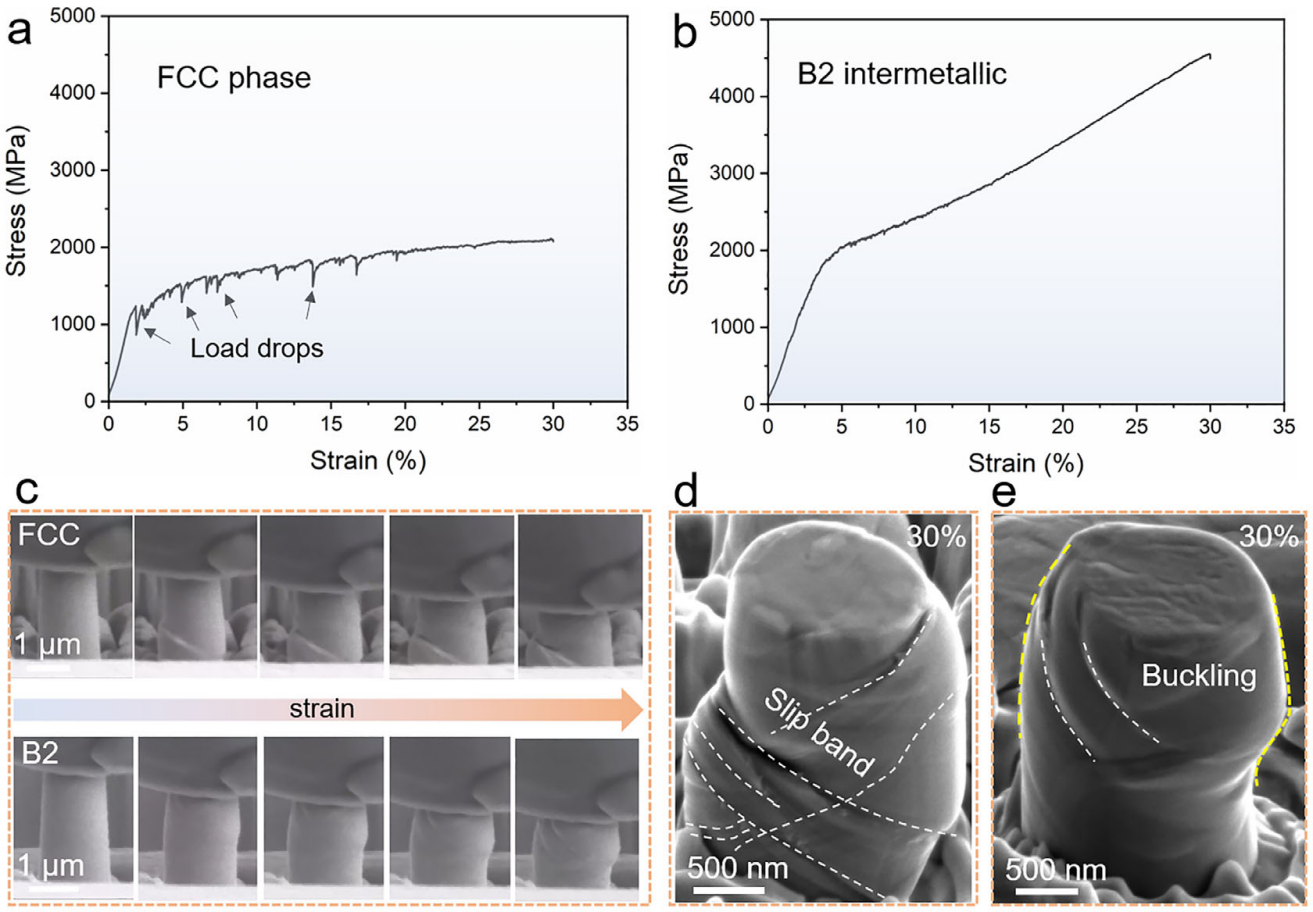
Micropillar compression behaviors of the FCC phase and B2 intermetallics. (a,b) Compressive stress–strain curves for the FCC and B2 micropillars. (c) Snaps of the micropillar deformed at different strains. (d) SEM images of an FCC micropillar after 30% strain, revealing the formation of pronounced slip bands. (e) SEM images of a B2 micropillar after 30% strain, displaying slight top‐surface bulging and shear‐band traces.

Uniaxial tensile tests of the DOIs alloy (Figure 3a) reveal an ultrahigh yield strength of 1.24 GPa, an ultimate tensile strength of 1.57 GPa, and a uniform elongation of 20% at room temperature. By comparison, both as‐cast (AC) and homogenization‐treated (HT) control alloys exhibit much lower yield strengths (0.81 and 0.73 GPa, respectively) and negligible ductility (Figure S6), underscoring the brittleness of their intermetallic phases. The DOIs alloy's fracture surface displays characteristic dimpled morphology (Figure 3b), further attesting to its excellent ductile behavior. In addition, the DOIs alloy exhibits a pronounced multi‐stage strain‐hardening response with a consistently stable strain‐hardening rate higher than 3 GPa across a wide strain range (Figure 3c), increasing the flow stress by approximately 0.33 GPa during plastic deformation. Noted that even at the strain of necking, the strain hardening rate can reach ∼1.87 GPa. This outstanding strain hardening considerably improves the strength and gives a large uniform elongation. When plotted against literature data (Figure 3d), our alloy surpasses conventional B2‐ordered alloys—typically brittle at ambient temperature [29, 30, 31]—as well as other FCC‐based alloys reinforced with hard brittle intermetallics [10, 20, 32, 33, 34, 35, 36, 37, 38, 39, 40, 41, 42, 43]. Although several recently reported alloys demonstrate even higher strength–ductility combinations [18, 19, 44], the current DOIs alloy still achieves an attractive balance of strength, ductility, and sustained strain hardening at room temperature.

**FIGURE 3:**
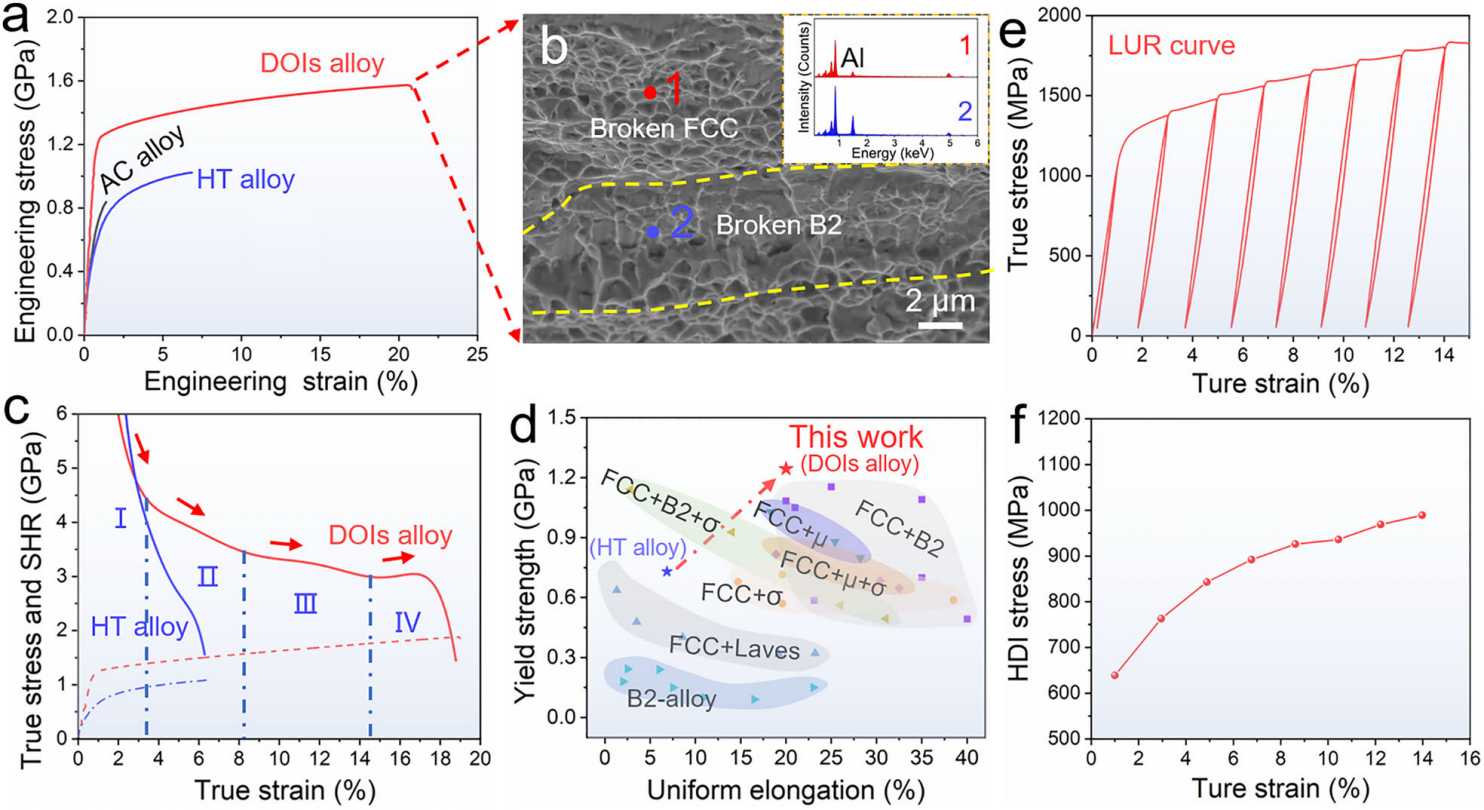
Tensile behavior and strain–hardening characteristics of the DOIs alloy at room temperature. (a) Engineering stress–strain curves comparing the DOIs alloy with AC and HT control alloys. (b) Fracture–surface SEM of the DOIs alloy, displaying ductile dimples. (c) True–stress (dashed line) and strain hardening rate (solid line) versus true strain; stages I–V denote distinct hardening regimes. (d) Plot of yield strength versus uniform elongation for the DOIs alloy against literature data for B2–ordered alloys and FCC–based MPEAs reinforced with hard brittle intermetallics [10, 19, 20, 29, 30, 31, 32, 33, 34, 35, 36, 37, 38, 39, 40, 41, 42, 43, 44]. (e) True stress–strain curves of LUR test. (f) The HDI stress evolution during plastic deformation of the DOIs alloy.

To better understand the origin of the observed high yield strength and strain hardening, we conducted loading‐unloading‐reloading (LUR) testing of the DOIs alloy, and the results are illustrated in Figure 3e. Pronounced hysteresis loops are observed within the true strain range of 0.01–0.14 that expand with increasing strain, confirming the activation of back stress. This long‐range stress arises from the accumulation of GNDs at interfaces such as grain boundaries, dislocation walls, and non‐coherent precipitates. The high density of such hetero‐interfaces in the DOIs alloy leads to a significant macroscopically measurable stress, known as HDI stress (σ_*HDI*_), which quantitatively captures the collective contribution of these back stresses. The HDI stress was quantified from the LUR hysteresis loops using the average value of the unloading yield stress (σ_μ_) and reloading yield stress (σ_*r*_), according to σ_*HDI*_ =  (σ_μ_ + σ_*r*_)/2 [45], as quantified in Figure 3f. The unloading and reloading yield points were determined by the deviation from linearity of the stress–strain curve. The magnitude and evolution of this stress confirm the potent deformation‐constraining effect revealed by prior microstructural characterization and establish HDI stress as the primary contributor to the high yield strength. The increase in HDI stress with plastic deformation results from progressive GND pile‐ups against hetero‐interfaces under escalating strain gradients. This behavior is enabled by the deliberately engineered heterogeneous microstructure, where hierarchical inhomogeneities spanning ultrafine to nanoscale interfaces generate extraordinary strain hardening. Together, the LUR results corroborate our microstructural observations and quantitatively establish sustained HDI stress and its associated hardening as the key mechanism responsible for the superior mechanical properties.

### 2.3 Microstructure Evolution of Deformed FCC and B2 Phases

To elucidate the deformation mechanisms responsible for the alloy's exceptional strength‐ductility synergy, we systematically characterized the substructural evolution of the dual‐phase DOIs alloy at incremental tensile strains (5%, 10%, and 16%) using bright‐field TEM under two‐beam condition.

At a low strain of 5%, the FCC phase demonstrates planar slip activity, characterized by dislocation pairs and nodes (Figure 4a), a signature deformation mode in FCC alloys with medium‐to‐high stacking fault energy (SFE) [2, 46]. In stark contrast, the B2 lamellae exhibit no statistically significant dislocation density increase compared to the undeformed state (Figure 4b), reflecting delayed plasticity initiation in the intermetallic phase. Upon reaching 10% strain, FCC dislocation arrays reorganize into high‐density dislocation walls (HDDWs) (Figure 4c), with intersecting slip planes generating dislocation tangles between the HDDWs. Concurrently, the B2 phase undergoes a significant increase in dislocation density relative to the 5% strain stage (Figure 4d), marking the onset of coordinated plasticity across both phases. As the strain increases to 16%, the FCC phase develops dense dislocation forests (Figure 4e), resulting from heightened dislocation multiplication and cross‐slip activity. Annealing twin boundaries further enhance strain hardening by impeding dislocation motion. Simultaneously, the B2 intermetallics establish homogeneous Taylor lattice structures (Figure 4f)—geometrically ordered dislocation networks formed via multi‐slip‐plane glide. These lattices, stabilized by high‐density stored dislocations, act as self‐generated barriers to subsequent dislocation motion [46, 47]. Collectively, these findings demonstrate that the alloy's mechanical superiority arises from spatially and temporally coordinated dislocation dynamics across phases, with glide‐mediated plasticity serving as the principal deformation mechanism.

**FIGURE 4:**
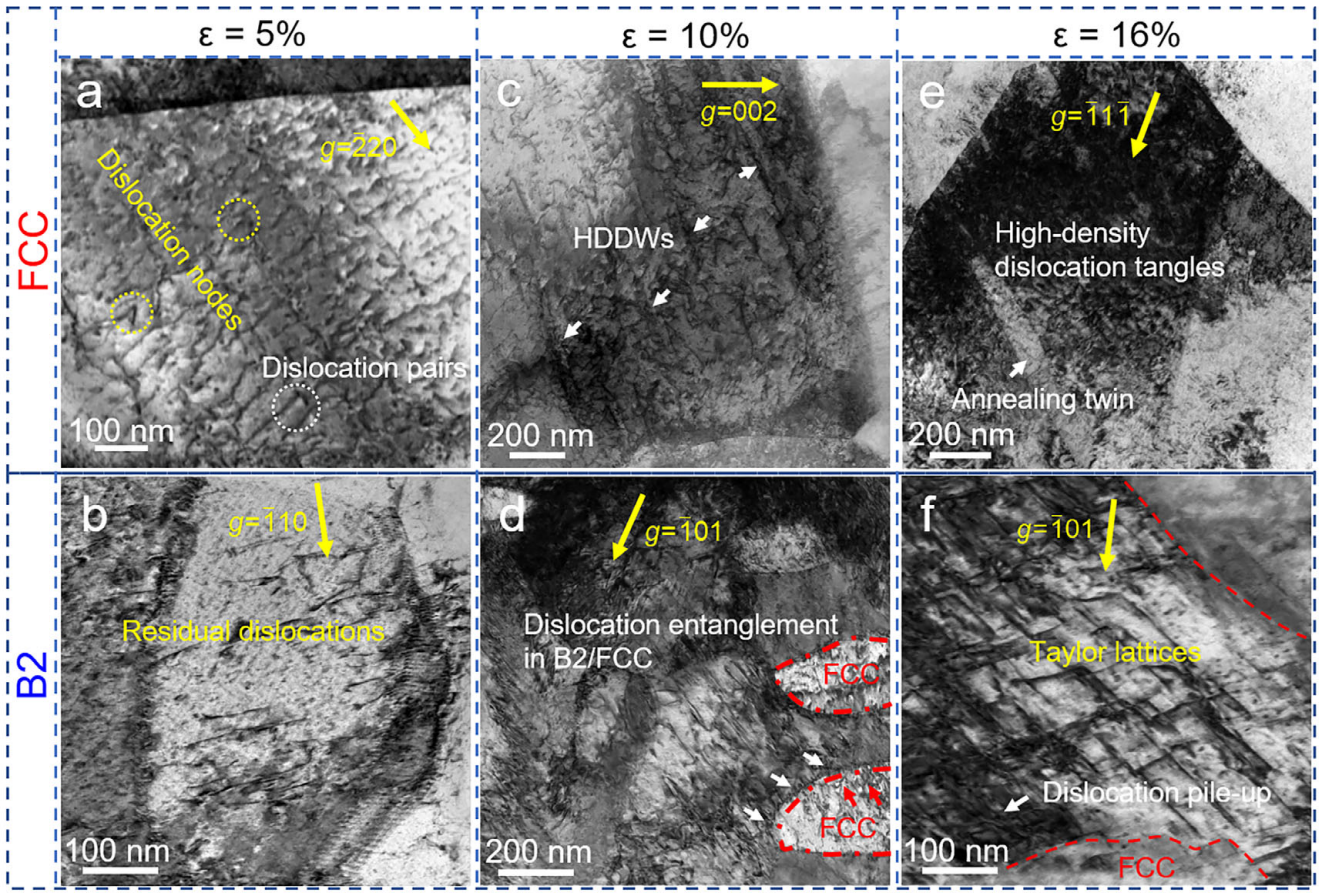
Evolution of dislocation substructures in the DOIs alloy with increasing true strain. (a,b) At ∼5% strain: (a) FCC lamellae exhibit planar slip, showing dislocation pairs and node formation; (b) B2 laths display no appreciable change in dislocation density. (c,d) At ∼10% strain: (c) FCC grains form high‐density dislocation walls with tangles at intersecting slip planes; (d) B2 laths show a threefold increase in dislocation density. (e,f) At ∼16% strain: (e) FCC regions develop dense, three‐dimensional dislocation forests; (f) B2 domains establish homogeneous Taylor lattices—ordered dislocation networks that act as dynamic barriers to further slip.

To directly visualize strain partitioning between the FCC and B2 phases during tensile deformation, in situ SEM tensile testing combined with digital image correlation (DIC) analysis was performed. Representative strain maps at different deformation stages are shown in Figure S7. At the early stage of plastic deformation, strain is preferentially localized within the FCC lamellae, indicating that the softer FCC phase accommodates most of the imposed strain. With increasing tensile strain, localized deformation progressively develops within the B2 domains, demonstrating the gradual activation of plasticity in the ordered intermetallic phase. At larger strains, both phases participate in deformation, leading to a more distributed strain field across the heterogeneous lamellar structure. These observations provide direct evidence for sequential plastic activation and coordinated co‐deformation between the FCC and B2 phases despite their substantial strength contrast.

Moreover, EBSD analysis near the fracture surface of the DOIs sample after tensile testing is presented in Figure S8. The grain morphology remained similar to the pre‐deformation microstructure shown in Figure 1. No deformation twins or phase transformation were observed in either the FCC or B2 laminated regions after tensile fracture. This absence of twinning was further confirmed by TEM and corresponding SAED pattern performed at the fracture strain (Figure S9), which provided direct diffraction evidence that mechanical twinning was not activated during deformation. While previous studies reported that multiple deformation twins can activate in such lamellae—enhancing ductility in ultrafine‐grained materials [48]—twinning was suppressed in the present case. This absence is attributed to the relatively high SFE and fine grain size of the FCC phase [49]. The high Ni (42.9 at.%) and V (22.8 at.%) contents, together with the absence of strong SFE‐lowering elements such as Co, suggest that the FCC phase possesses a moderate‐to‐high SFE compared with typical twinning‐induced plasticity alloys [50, 51, 52]. Instead, a significant increase in local misorientations was observed (Figure S8c), indicating a higher density of GNDs and more pronounced dislocation activity following tensile deformation. As shown in Figure S8d, the KAM value of the B2 intermetallics in the fractured DOIs sample increased from 0.64° to 1.32°. This rise in GND density with plastic strain enhanced strain hardening in the B2 phase, consistent with the TEM observations in Figure 4.

## 3 Discussion

### 3.1 Origin of Ductile B2 Intermetallics

The inherent brittleness of conventional intermetallic compounds often leads to stress concentrations, crack initiation, and limited ductility [29, 31, 32]. In stark contrast, our DOIs alloy contains a high‐volume fraction of B2 intermetallics (52 vol.%) that exhibit pronounced plasticity, enabling an exceptional strength–ductility synergy. Three key design principles underlie this transformation. First, shifting from a simple NiAl composition to a multicomponent Ni(Al,Fe,V) B2 phase reduces anti‐phase boundary energy and chemical ordering energy, thereby facilitating dislocation activation compared to the binary NiAl [1, 20]. Second, nanoscale chemical fluctuations—stemming from incomplete element partitioning—create atomic‐scale heterogeneities that impede dislocation motion, increasing dislocation interactions and amplifying strain hardening within the B2 domains [53]. Third, the high density of pre‐existing dislocations in the ordered phase facilitates further dislocation multiplication under load. These factors together convert the B2 intermetallics from a brittle crack nucleator into a robust, ductile constituent, whose pronounced work hardening delays necking and enhances overall alloy ductility.

### 3.2 Formation of Heterogeneous Lamellar Structure

Beyond endowing the B2 phase with intrinsic ductility, engineering a mesoscale laminated morphology is crucial to the alloy's mechanical performance. As illustrated schematically in Figure S10, the as‐cast Ni_2_FeVAl_0.5_ alloy exhibits a dual‐phase microstructure comprising FCC and B2 phases, with fine FCC islands distributed along the interdendritic regions and B2 grain boundaries (Figure S11a,b). After homogenization, this dual‐phase morphology is largely retained, although the FCC islands show slight aggregation (Figure S11c,d). A 90% cold‐rolling reduction then stretches and thins the primary FCC grains and eutectic FCC/B2 bands into a severely deformed lamellar stack (Figure S12). Detailed TEM (Figure S13) shows that the B2 regions accumulate a high density of dislocations and slip bands during rolling, while the FCC bands evolve into a nanolamellar structure that promotes recovery and recrystallization upon annealing [16].

Upon annealing at 850°C, the heavily deformed FCC lamellae fully recrystallize into ultrafine, equiaxed grains, as confirmed by EBSD phase and image quality maps showing fine FCC grains with high‐angle boundaries (Figure S3). Concurrently, Ni–Al supersaturation drives precipitation of near‐equiaxed B2 particles along their boundaries. These B2 precipitates exert a strong Zener‐pinning effect, suppressing FCC grain growth and stabilizing an ultrafine‐grained, dual‐phase lamella within the former FCC bands. In contrast, the cold‐worked B2 lamellae predominantly undergo recovery, evidenced by low‐angle subgrain boundaries (Figure S14), retained dislocations, and elevated local misorientations in B2‐rich regions in the KAM map (Figure S3d). Although the misorientation distribution of the B2 phase indicates the presence of a limited number of high‐angle boundaries, suggesting minor local recrystallization, recovery remains the dominant mechanism. This behavior can be attributed to the intrinsically sluggish recrystallization kinetics of the multicomponent Ni(Al,Fe,V) B2 phase. Compared with conventional B2 intermetallics, the chemically complex B2 phase exhibits severe lattice distortion and pronounced local compositional fluctuations, both of which impede defect rearrangement and retard dislocation annihilation during annealing. In addition, the incorporation of the high‐melting‐point V element enhances the thermal stability of the B2 phase, further suppressing complete recrystallization. Driven by V and Fe supersaturation (Figure S13b), this recovery process promotes the nucleation of ultrafine FCC grains along slip‐band networks within the B2 [16]. The result is an alternating stack of ultrafine FCC layers and ductile B2 laths.

Finally, incomplete partitioning during recovery gives rise to nanoscale chemical heterogeneities within the B2—atomic‐scale composition fluctuations that further impede dislocation motion and bolster strain hardening. Together, this hierarchical lamellar architecture orchestrates strain partitioning and stress redistribution, effectively suppressing interfacial cracking and unlocking the alloy's exceptional combination of high strength and large ductility.

### 3.3 Effects of Microstructure Heterogeneity on the Deformation Behavior

To elucidate the plastic mismatch and heterogeneous deformation in the DOIs alloy, nanoindentation tests were conducted across different regions of the specimen (Figure S15). The corresponding EBSD phase map (Figure S15b) was used to identify three typical regions: the FCC layers, the FCC/B2 interface, and the B2 layers. Enlarged views of three selected positions (Figure S15c1–c3) show clearly visible slip lines in the FCC layers. However, these slip lines are barely visible in the B2 layers, even though some FCC phase is present beneath the indent at position c3, which is consistent with the micropillar compression behaviors in Figure 2. Additionally, the load–displacement curves at positions c2 and c3 show smooth, continuous responses, whereas that at position c1 exhibits a distinct pop‐in event between 83 and 88 nm, corresponding to slip‐line formation in the FCC layers. Specifically, the pop‐in occurs in regions with low dislocation density—such as the recrystallized FCC layers at position c1—due to the abrupt nucleation of new dislocation loops [54]. In contrast, continuous deformation was observed in regions with high dislocation density—such as the B2 layers at position c3—driven by conventional Frank–Read dislocation multiplication [54]. Furthermore, the B2 layers exhibited the highest nanohardness value of 7.17 GPa, compared to 6.50 GPa at the FCC/B2 interface and 5.67 GPa in the FCC layers. Statistical analysis of nanohardness distribution (Figure S15d) further confirms that the B2 layers are the dominant contributor to the overall strengthening of the DOIs alloy, owing to its high nanohardness (∼7.11 GPa).

Plastic deformation in the DOIs alloy proceeds through sequential activation of dislocations in the alternating soft FCC and hard B2 regions, giving rise to a pronounced multistage strain‐hardening response and a high strain‐hardening rate (> 3 GPa) (Figure 3c). This sequential deformation process is further evidenced by the in situ DIC strain maps (Figure S7), which show that strain initially localizes within the softer FCC lamellae and progressively extends into the harder B2 domains with increasing tensile strain. In stage I, immediately after yielding, this hardening rate drops sharply because the initial mobile dislocation density is insufficient to accommodate plastic flow [19, 24]. Deformation is carried almost exclusively by the softer FCC layers, where early slip activation produces limited hardening. As the strain increases into Stage II, dislocation multiplication and planar glide in the FCC phase gradually replenish mobile dislocations, yielding a more moderate decline in strain‐hardening rate. Although stresses remain below the critical level needed to nucleate new dislocations in the B2 intermetallics, pre‐existing dislocations within the B2 can still glide, contributing modestly to work hardening. Once the true strain approaches ∼8%–14% (Stage III), the B2 domains activate robust dislocation sources. Co‐deformation of FCC and B2 regions causes intense dislocation accumulation at their interfaces, curtailing the mean free path of mobile dislocations and dramatically boosting the rate of strain hardening. The hard B2 laths constrain the softer FCC layers, leading to high back stresses (Figure 3f) and GND pile‐ups that further elevate work hardening. Beyond ∼16% strain, both phases activate additional slip systems, leading to intensified dislocation activity. In the FCC lamellae, these manifest as dense three‐dimensional dislocation tangles, while in the B2 laths, ordered Taylor lattices of highly stored dislocations form (Figure 4e,f). This concurrent buildup invokes a dynamic Hall–Petch effect [47], sustaining a high hardening rate. After reaching its peak, the rate of strain hardening gradually declines. At this stage, the mutual annihilation of gliding dislocations and the onset of cross‐slip [2] reduce the net mobile dislocation density, curtailing further hardening and signaling the approach of plastic instability (necking). This orchestrated sequence of dislocation activation, storage, and interaction underpins the DOIs alloy's extraordinary combination of ultrahigh strength and large uniform elongation.

### 3.4 Crack Propagation Resistance

Another intriguing finding of this study is the unique fracture behavior induced by the structural design of the DOIs alloy. Owing to the stiffness mismatch between hard B2 laths and softer FCC layers, stress concentrations develop at their interfaces, providing preferential sites for microcrack nucleation when dislocation pile‐ups exceed the local decohesion threshold [55]. These microcracks initiate at the FCC/B2 boundaries and propagate into the lamellae, where coalescing voids give rise to discontinuous cracks (Figure 5a–c). Crucially, the ductile B2 intermetallics dispersed within the FCC regions blunt advancing crack tips and impede further crack growth, delaying catastrophic failure [55]. Conversely, the softer FCC grains embedded in the B2 lamellae locally relax stresses through plastic deformation, promoting crack deflection and arrest within the B2 lamellae (Figure 5d–f). Additionally, the nanoscale chemical heterogeneities and dense residual dislocations inside the B2 domains further elevate their toughness and crack‐arrest capabilities [36]. Together, this combination of hard‐yet‐deformable B2 laths and ductile FCC layers in a layered architecture provides an effective strategy for superior fracture resistance in high‐strength alloys.

**FIGURE 5:**
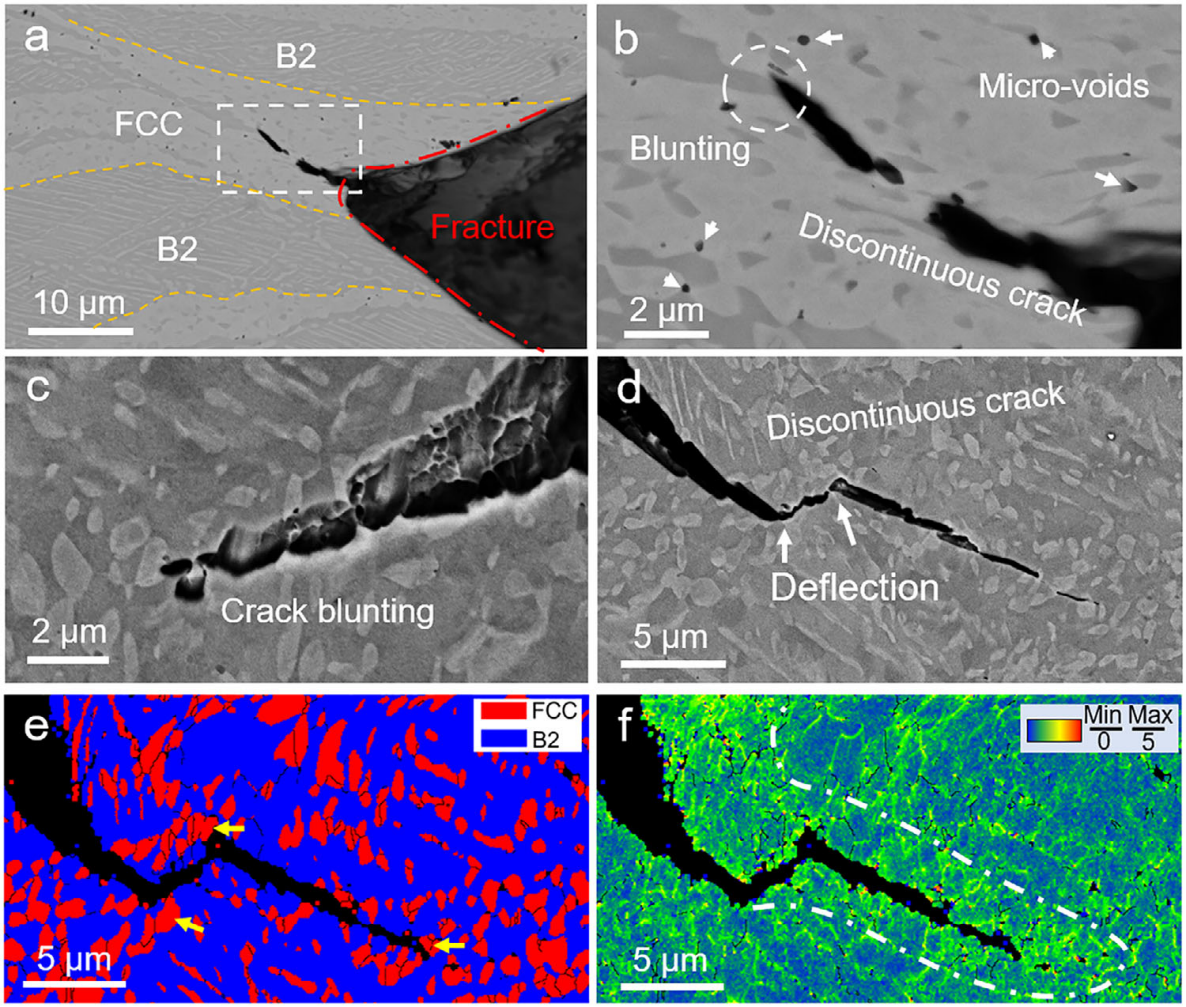
Crack–propagation behavior in the DOIs alloy. (a,b) SEM of crack initiation and void formation in the FCC‐laminated region; B2 laths blunt advancing cracks. (c,d) SEM of crack deflection and blunting within the B2‐laminated region. (e,f) EBSD phase map (e) and KAM map (f) of the B2‐rich area, illustrating crack tip deflection along phase boundaries.

## 4 Conclusions

In summary, we introduce a design strategy that combines intrinsically ductile, multicomponent B2 intermetallics with a finely engineered, dual‐phase lamellar architecture to achieve an exceptional combination of mechanical performance—a yield strength of 1.24 GPa, an ultimate tensile strength of 1.57 GPa, and 20% uniform elongation, accompanied by a high strain hardening rate exceeding 3 GPa across a wide strain range. By tuning the B2 to include Ni, Al, Fe, and V, and by harnessing nanoscale compositional fluctuations and a high density of pre‐existing dislocations, we activate extensive dislocation multiplication within the ordered phase to overcome its conventional brittleness. Sequential activation of dislocation sources in the soft FCC layers and the hard B2 laths produces pronounced multistage strain hardening. This sustained hardening delays the onset of necking and synergistically enhances both strength and ductility. Finally, the alternating soft‐hard lamellae synergistically blunt and arrest cracks—ductile B2 laths blunt cracks in the FCC regions, while compliant FCC laminates relieve stress at crack tips in the B2 regions—further enhancing damage tolerance. This paradigm of “ductile intermetallics plus mesoscale heterogeneity” opens a fascinating new pathway for designing next‐generation structural alloys that no longer trade strength for ductility.

## 5 Materials and Methods

### 5.1 Alloy Fabrication

The Ni_2_FeVAl_0.5_ alloy was prepared via electromagnetic levitation melting under a high‐purity argon atmosphere using raw pure metals (≥ 99.9 wt.% purity). A 1.5 kg ingot was cast, homogenized at 1150°C for 5 h under argon, and water‐quenched to minimize elemental segregation. The homogenized alloy was cold‐rolled in 8 passes to achieve a 90% thickness reduction. Post‐rolling sheets were vacuum‐sealed in quartz tubes, annealed at 850°C for 5 h, and water‐quenched to retain room‐temperature microstructures.

### 5.2 Mechanical Testing

Dog‐bone‐shaped tensile specimens (gauge dimensions: 10 mm × 2.0 mm × 0.5 mm) were wire‐cut from annealed sheets using electrical discharge machining. Gauge surfaces were mechanically polished to 1200‐grit SiC paper. Uniaxial tensile tests and LUR tests were performed at room temperature on a servo‐hydraulic testing machine (MTS CMT5105) with a strain rate of 1 × 10^−3^ s^−1^. Strain evolution was tracked via stereo digital image correlation (DIC) by analyzing displacements of a speckle pattern sprayed on the gauge surface. Reported tensile properties reflect the average of three independent tests.

In situ micropillar compression tests were conducted in a scanning electron microscope (SEM, MIRA 3, TESCAN) equipped with an FT‐NMT04 nanomechanical tester. Micropillars (Φ = 1 µm, aspect ratio 2.5) were fabricated site‐specifically using a focused ion beam (FIB, FEI Helios NanoLab 600i). To ensure that each pillar consisted solely of either the FCC or B2 phase, large individual grains (> 2 µm) were first identified based on their distinctive SEM contrast. During FIB milling, the cross‐section of each pillar was continuously monitored to verify a uniform single‐phase structure. Compression tests were performed using a diamond flat‐punch tip (Φ = 10 µm) at a constant strain rate of 5 × 10^−4^ s^−^^1^. Three independent tests were conducted for each phase, with force–displacement data recorded and real‐time videos captured.

In situ micro‐tensile tests were performed in a SEM (Thermo Scientific Apreo 2C) equipped with an in situ tensile testing stage (HS4000) at a constant displacement rate of 0.1 mm/min. Strain localization and distribution during uniaxial tensile deformation were quantified using DIC analysis. Dog‐bone‐shaped specimens with a gauge section of 2 mm × 1.5 mm × 0.5 mm were used for SEM‐DIC observations, and raw data analysis was performed using ViC‐2D software.

### 5.3 Microstructural and Compositional Characterization

Phase identification was performed via XRD (Rigaku SmartLab‐9 kW) with Cu Kα radiation (λ = 1.54056 Å). The XRD scans were conducted with a scan step size of 0.02° over a 2θ range of 20°‒100°. The alloy's grain morphology, orientation, and size were analyzed using EBSD (Nordlys Max^2^, Oxford). EBSD specimens were initially polished with SiC paper to 1200 grit, followed by vibrational polishing in colloidal silica suspension for 12 h. The alloy's microstructures and compositions were characterized by TEM (FEI Talos F200X) operated at 200 kV. TEM specimens were prepared by FIB milling using the standard lift‐out technique. Geometric phase analysis of high‐resolution TEM images was conducted using FRWRTools script. Strain maps were plotted with respect to an internal reference lattice by g_1_ = (101) and g_2_ = (110) for the B2 phase using Lorentzian masks (in reciprocal space). The fracture surfaces were examined by a field‐emission SEM (TESCAN MIRA 3, Czech Republic) equipped with an EDS detector. APT needles were FIB‐fabricated and analyzed in a CAMECA LEAP 5000 XR. APT data reconstruction and analysis utilized Imago Visualization and Analysis Software (IVAS v3.8).

## Author Contributions

L.Y. fabricated the alloy, carried out the majority of experiments, prepared the figures, and drafted the manuscript. L.Y., F.L.J., Q.M.Z., D.S.L., J.S.L., and K.J.C. analyzed the data. J.H.L. and Z.B.J. performed the APT characterization. F.Z.R. conceived and supervised the project, analyzed the data, and revised the manuscript. R.O.R. analyzed the data and revised the manuscript. All authors contributed to the discussion of the results and provided feedback on the manuscript.

## Conflicts of Interest

The authors declare no conflict of interest.

## Supporting information




**Supporting File 1**: advs76697‐sup‐0001‐SuppMat.docx.

**Supporting File 2**: advs76697‐sup‐0002‐VideoS1.mp4.

**Supporting File 3**: advs76697‐sup‐0003‐VideoS2.mp4.

## Data Availability

The data that support the findings of this study are available from the corresponding author upon reasonable request.
