# Supplementary material for: Ductile B2 Intermetallics‐Driven Strength‐Ductility Synergy in Heterolaminated Multi‐Principal Element Alloys
Source: Adv Sci (Weinh). 2026 Jul 24;13(57):e76697. doi: 10.1002/advs.76697 (PMC13398132; doi:10.1002/advs.76697)
Supplement: Supplementary file 1 [file ADVS-13-e76697-s002.docx]

**Supplementary Information for**

**Ductile B2 intermetallics-driven strength-ductility synergy in heterolaminated multi-principal element alloys**

Lu Yang^1^, Feilong Jiang^1^, Qiming Zhuang^1^, Dingshan Liang^1^, Jiasi Luo^1^, Kangjie Chu^1^, Junhua Luan^2^, Zengbao Jiao^3^, Robert O. Ritchie^4, *^, Fuzeng Ren^1, *^

^1^Department of Materials Science and Engineering, Southern University of Science and Technology, Shenzhen, Guangdong 518055, China

^2^Department of Materials Science and Engineering, City University of Hong Kong, Hong Kong 999077, China

^3^Department of Mechanical Engineering, The Hong Kong Polytechnic University, Hong Kong 999077, China

^4^Department of Materials Science and Engineering, University of California, Berkeley, CA 94720, USA

*Corresponding Authors. Email: [renfz@sustech.edu.cn](mailto:renfz@sustech.edu.cn) (F.R.); [ritchie@berkeley.edu](mailto:ritchie@berkeley.edu) (R.O.R.)

**This PDF file includes:**

Supplementary Text

Figures S1 to S15

Legends for Movies S1 to S2

References

**Other supporting materials for this manuscript include the following:**

Movies S1 to S2

**Supplementary Text**

*Alloy design and material processing*

A Ni_2_FeVAl_0.5_ alloy with an actual composition of Ni_43.4_Fe_22.7_V_21.3_Al_12.6_ (at %) was designed. Vanadium was selected for its pronounced atomic size misfit, inducing severe lattice distortion and elastic strain fields to enhance solid-solution strengthening [1, 2]. To stabilize an FCC matrix, the Ni_2_FeV base composition was tuned to a valence electron concentration (VEC) of 8.25, exceeding the critical VEC value (~8.0) for FCC phase dominance in MPEAs [3]. The addition of Al element promotes the transition from FCC to BCC/B2 phase, forming a dual-phase microstructure. Thus, FCC Ni_2_FeV served as the base alloy, with Al element added to reduce the VEC and induce ordered B2 phase precipitation. Additionally, the Al solutes, which have a large negative mixing enthalpy with Ni, Fe, and V, produce local chemical inhomogeneities within the B2 phase. Phase stability was validated via CALPHAD (Calculation of Phase Diagrams) method with the TCHEA6 database. The calculated phase diagram of the (Ni_2_FeV)-Al system is presented in Fig. S1a. The diagram indicates that the FCC phase forms first as the temperature decreases, followed by the Ni_2_FeVAl_0.5_ alloy entering the FCC+B2 two-phase region below 1300 °C.

The heterogeneous lamellar structure was engineered through controlled thermomechanical treatments, involving a 90% cold-rolling reduction followed by annealing at 850 °C for 5 h (Fig. S1b). The heavy cold-rolling induces severe plastic deformation in the FCC and B2 phases, resulting in the formation of the laminated structure. The FCC phase undergoes full recrystallization, while the B2 phase only enables recovery, leaving a high density of residual dislocations and partial homogenization within the B2 phase during subsequent thermal treatment [4].

**Fig. S1.** Thermodynamic phase diagram calculation and preparation process of Ni_2_FeVAl_0.5_ alloy. (a) Phase diagram of the (Ni_2_FeV)_1-_*_x_*Al*_x_* (at.%) system, calculated by the Thermo-Calc software with the TCHEA6 database. (b) Schematic illustration of the thermomechanical process used to produce the alloy in this work.

**Fig. S2.** The non-uniform distribution of local misorientations in the DOIs alloy. (a) KAM map. (b) The statistical results of corresponding KAM value.

**Fig. S3.** The inhomogeneous distribution within the FCC-rich and B2-rich layers. (a) EBSD image quality map. (b) IPF map. (c) Phase map. (d) KAM map.

**Fig. S4.** Microstructure analysis of the FCC laminated regions prior to tensile deformation. (a) HAADF-STEM analysis, the insert image showing the selected site of FCC laminated regions. (b) SAED and HRTEM image of FCC phase showing annealing twins. (c) SAED and HRTEM image of B2 phase; (d) Corresponding EDS elemental maps of Al, V, Ni and Fe. (e) The corresponding EDS line profile across the yellow arrow marked in a, showing the partition of constitutive elements between FCC and B2 phases.

**Fig. S5.** Frequency distribution of Ni, Fe, V, and Al atoms within the B2 phase, compared with the ideal binomial distribution (dashed line) for a random solid solution. The observed deviations from the binomial distribution indicate the presence of significant nanoscale chemical fluctuations. The normalized homogenization parameters (μ) are substantially higher than values reported for homogeneous solid solutions [5-7], confirming the non-random elemental distribution in the B2 phase.

**Fig. S6.** The fracture surfaces of the AC and HT alloys with typical cleavage fractures, indicating their brittle nature. (a) AC alloy. (b) HT alloy.

**Fig. S7.** In situ DIC strain maps of the DOIs alloy at different tensile strains, illustrating strain partitioning and deformation coordination between the FCC and B2 phases.

**Fig. S8.** EBSD characterization of the fractured DOIs sample. (a) IPF image. (b) Phase image. (c) KAM image. (d) The statistical results of corresponding KAM value.

**Fig. S9.** (a) TEM image and (b) corresponding SAED pattern of the FCC regions at fracture strain. Dislocations within the FCC phase are pinned by B2 nanoparticles, and no mechanical twinning is observed.

**Fig. S10.** Schematic illustration of the formation mechanism of the multi-heterogeneous lamellar structure.

**Fig. S11.** Microstructures of the as-cast and homogenized Ni_2_FeVAl_0.5_ alloy. (a, b) EBSD IPF and corresponding phase maps of the as-cast alloy. (c, d) EBSD IPF and phase maps of the homogenized alloy, showing retention of the dual-phase morphology.

**Fig. S12.** SEM-EDS characterization of the cold-rolled sample. (a-c) SEM images at different magnifications; (d) The corresponding EDS line profile of Al element across the blue and red dashed line marked in b and c, respectively, showing the partition of Al element between FCC and B2 phases.

**Fig. S13.** TEM characterization of the cold-rolled sample. (a) Bright-field TEM image showing the distribution of deformed B2 and FCC bands. (b) The corresponding STEM-EDS elemental maps of (a). (c) The high-magnification bright-field TEM image of B2 phase showing a high density of slip bands. (d) The high-magnification bright-field TEM image of FCC phase showing the formation of nanolaminated structure.

**Fig. S14.** The misorientation angle distribution of FCC and B2 phase in the DOIs alloy. The FCC phase has high-density annealing twin boundaries, indicating the occurrence of recrystallization. However, the B2 phase contains high-density low angle grain boundaries, indicating recovery rather than recrystallization.

**Fig. S15.** Nanoindentation analysis of the DOIs alloy. (a-b) Low-magnification SEM image and corresponding EBSD phase map showing the distribution of indents. The inserted white square are the selected regions marked by positions c1-c3 in (a). (c) The enlarged areas represent the c1: FCC layers, c2: FCC/B2 interface and c3: B2 layers. Their corresponding load-displacement curves are also displayed. (d) Statistical distribution of nanohardness of three regions including FCC layers, FCC/B2 interface and B2 layers.

**Movie. S1 (separate file). In situ compression tests of FCC phase micropillar.**

**Movie. S2 (separate file). In situ compression tests of B2 phase micropillar.**

**References**

[1]	S. S. Sohn, A. Kwiatkowski da Silva, Y. Ikeda, F. Kormann, W. Lu, W. S. Choi, B. Gault, D. Ponge, J. Neugebauer, D. Raabe, Ultrastrong medium-entropy single-phase alloys designed via severe lattice distortion, Adv Mater. 11 (2018) e1807142.

[2]	B. Yin, F. Maresca, W.A. Curtin, Vanadium is an optimal element for strengthening in both fcc and bcc high-entropy alloys, Acta Mater. 188 (2020) 486-491.

[3]	S. Guo, C. Ng, J. Lu, C.T. Liu, Effect of valence electron concentration on stability of fcc or bcc phase in high entropy alloys, J. Appl. Phys. 109 (2011) 103505.

[4]	Q. Wu, F. He, J. Li, H.S. Kim, Z. Wang, J. Wang, Phase-selective recrystallization makes eutectic high-entropy alloys ultra-ductile, Nat. Commun. 13 (2022) 4697.

[5]	F. Wang, M. Song, M.N. Elkot, N. Yao, B. Sun, M. Song, Z. Wang, D. Raabe, Shearing brittle intermetallics enhances cryogenic strength and ductility of steels, Science 384 (2024) 1017-1022.

[6]	J. Zhou, Q. Wang, Q. Zeng, K. Yin, A. Wang, J. Luan, L. Sun, B. Shen, A plastic FeNi-based bulk metallic glass and its deformation behavior, J. Mater. Sci. Technol. 76 (2021) 20-32.

[7]	C.C. Yuan, Z.W. Lv, C.M. Pang, X.L. Wu, S. Lan, C.Y. Lu, L.G. Wang, H.B. Yu, J.H. Luan, W.W. Zhu, G.L. Zhang, Q. Liu, X.-L. Wang, B.L. Shen, Atomic-scale heterogeneity in large-plasticity Cu-doped metallic glasses, J. Alloys Compd. 798 (2019) 517-522.
